# Gene Families With Stochastic Exclusive Gene Choice Underlie Cell Adhesion in Mammalian Cells

**DOI:** 10.3389/fcell.2021.642212

**Published:** 2021-04-29

**Authors:** Mikhail Iakovlev, Simone Faravelli, Attila Becskei

**Affiliations:** Biozentrum, University of Basel, Basel, Switzerland

**Keywords:** allelic exclusion, carbonic anhydrase, cell identity, Poisson-binomial distribution, single-cell RNA-seq, basigin, olfactory receptor, mouse

## Abstract

Exclusive stochastic gene choice combines precision with diversity. This regulation enables most T-cells to express exactly one T-cell receptor isoform chosen from a large repertoire, and to react precisely against diverse antigens. Some cells express two receptor isoforms, revealing the stochastic nature of this process. A similar regulation of odorant receptors and protocadherins enable cells to recognize odors and confer individuality to cells in neuronal interaction networks, respectively. We explored whether genes in other families are expressed exclusively by analyzing single-cell RNA-seq data with a simple metric. This metric can detect exclusivity independently of the mean value and the monoallelic nature of gene expression. Chromosomal segments and gene families are more likely to express genes concurrently than exclusively, possibly due to the evolutionary and biophysical aspects of shared regulation. Nonetheless, gene families with exclusive gene choice were detected in multiple cell types, most of them are membrane proteins involved in ion transport and cell adhesion, suggesting the coordination of these two functions. Thus, stochastic exclusive expression extends beyond the prototypical families, permitting precision in gene choice to be combined with the diversity of intercellular interactions.

## Introduction

The combinatorial principle plays an important role in the evolution of complex organisms. A large proportion of the mammalian genomes encodes regulators, especially transcription factors (Vaquerizas et al., 2009), which determine what combination of genes will be turned on and off. Each cell type expresses a distinct set of genes, a form of phenotypic diversity that has been studied by single cell expression profiling, such as single-cell RNA-seq, with an unprecedented throughput (Baran-Gale et al., 2018). The study of the combinatorial expression patterns of genes belonging to a gene family or gene array is of particular relevance, among which the exclusive gene choice of the odorant and T-cell receptors has received widespread attention.

Each olfactory neuron expresses a single odorant receptor isoform randomly selected from more than a thousand gene isoforms (Massah et al., 2015; Khamlichi and Feil, 2018) and triggers a signal in response to a particular odor. Thus, precision of expression in a single cell is combined with diversity in a cell population. A similar principle underlies the immune response: each lymphocyte expresses a single antigen receptor randomly chosen from a large repertoire. The receptor isoforms are diversified, in part, due to the stochastic gene choice of the variable domain. With the in-depth study of these systems, it became apparent that a non-negligible proportion of cells expresses more than one, typically two gene isoforms (Brady et al., 2010). These cells with dual T cell receptors may enhance the antiviral response but can also underlie autoimmune disorders (Ji et al., 2010; Bradley et al., 2017). Thus, stochastic gene choice has clear physiological implications.

A slightly different form of exclusivity was observed in the protocadherin (Pcdh) array, which encodes multi-subunit membrane proteins mediating cell-to-cell interactions between neurons (Yagi, 2012). In this array, most cells express at least two distinct variable α-isoforms from a repertoire of 12 genes, one from the paternal, one from the maternal chromosome (Esumi et al., 2005). These findings indicate that the strict definition of exclusivity—one gene (isoform) per single cell—needs extending to account for the observed distributions and for averages greater than one.

These observations lead to the question about how to define exclusive expression in terms of a probability distribution. Is the expression of T-cell receptor isoforms exclusive if cells with dual T-cell receptors constitute 1, 50, or 90% of the population? What if three different receptor isoforms were to be expressed in some of the cells (Vatakis et al., 2013)? Recently, the degree of exclusivity in the stochastic gene choice of the Pcdh gene array was quantified with a probabilistic approach that defines exclusivity independently of the mean number of expressed genes in an array (Wada et al., 2018). This definition of stochastic exclusivity implies that the distribution of the number of expressed gene isoforms is narrower than expected from the purely random, independent expression of the genes in the array. For example, gene choice is precise when the majority of cells express three gene isoforms and only a few cells express less or more than three isoforms. Thus, stochastic exclusivity reflects simply the precision in gene choice irrespective of the underlying mechanism, let it be chromosomal looping during gene activation, negative feedback or allelic exclusion after DNA recombination.

Here, we examined single-cell RNA-seq data and established the exclusivity in the classic gene arrays and families, the odorant receptors, the T-cell receptors and the Pcdh-α array in some cell types, with a simple metric, regardless of whether gene expression is monoallelic or has a mean value of one. After this validation of our approach, we examined how the genome-wide organization of the genes affects stochastic gene choice and detected gene families (paralogs) with exclusive gene choice.

## Results

### Single Cell RNA-Seq Datasets

We analyzed RNA-seq datasets consisting of at least 100 single cell measurements of a well-defined cell type isolated from the mouse *Mus musculus*. Neurons from various locations in the nervous system were included, such as somatosensory neurons from dorsal root ganglions (Li et al., 2016), dopaminergic neurons (Hook et al., 2018) and corticostriatal neurons from the visual cortex (Tasic et al., 2016). Non-neuronal cell types encompassed nearly all organs: two types of lymphocytes, CD8^+^ T-cells (Kakaradov et al., 2017) and type 17 helper cells (Th17) (Gaublomme et al., 2015); dendritic cells from the bone marrow (Schlitzer et al., 2015), cardiomyocytes (Nomura et al., 2018), endothelial cells (Veerman et al., 2019), enterocytes (Haber et al., 2017), fibroblasts (Reinius et al., 2016), kidney duct cells (Chen et al., 2017), thymus epithelial cells (Sansom et al., 2014), prostate stromal cells (Kwon et al., 2019), type I and II alveolar cells from the lung (Guo et al., 2019); hepatoblasts and hepatocytes from the liver (Yang et al., 2017), pancreatic endocrine cells (Yu et al., 2019). Undifferentiated cell types were represented by embryonic stem cells isolated from embryos (Cheng et al., 2019) and embryonic stem cell (ESC) cultures (Klein et al., 2015). The gene expression has UMI units in two studies, while all other studies have FPKM/TPM units (Supplementary Table 1). The libraries in most studies were generated by Smart-Seq2 or its variants, which typically capture more genes than other technologies (Baran-Gale et al., 2018).

### Dichotomization of RNA-Seq Counts

The distribution of the RNA counts in a single-cell RNA-seq dataset is determined by various factors, in particular, the stochastic processes in gene expression and the methods for amplifying and detecting the RNA molecules. Gene expression is stochastic due to the low copy number of genes and mRNA molecules, and due to the spatiotemporal nature of biochemical processes in the cell (Battich et al., 2015; Baudrimont et al., 2019; Finn and Misteli, 2019; Friedrich et al., 2019; Rodrigo, 2019). When the expression has two states (OFF and ON states), the resulting distribution can be bimodal, often referred to as stochastic gene choice. Many genes display bimodal expression (Supplementary Figure 1A; Shalek et al., 2013).

To determine the proportion of OFF and ON cells, the RNA distribution must be dichotomized. For this purpose, we compared two classes of methods. In the moment-based methods, the averages or variances of the total distribution or parts of it are calculated. The second class of methods relies on the fitting of probability mass or density functions (pdf). The moment-based methods are more robust but lack a uniform mathematical framework (Supplementary Figures 1B–D). Conversely, the pdfs have mathematically well-defined dichotomization points but their fitting is less robust. In order to combine the advantages of the two approaches, we aimed at selecting the moment based approach that correlates the most with the dichotomization using pdfs.

We tested three types of moment-based methods: the Variance Reduction Score (VRS), Fraction of Maximal values (FM) and Geometric Trimmed Mid-Extreme threshold (GTME) (section “Materials and Methods”). The VRS quantifies the extent to which a given threshold reduces the sum of the variances of the two subpopulations relative to the unsplit population (Hellwig et al., 2010). The threshold minimizing the VRS was selected for the dichotomization. We devised two additional methods based on biological control principles, the FM and GTME. The FM is based on the assumption that a biological function can be performed as long as a variable in the ON state does not deviate too much from an optimal level. Accordingly, we defined the FM-threshold as the one tenth of the observed maximal values in the distribution. The GTME threshold is the geometric mean of the extreme values of the distribution; thus, it combines information on both the minimal and maximal values of the distribution.

To find the appropriate distribution, the specific pdf was selected in an unbiased way from a large number of known probability mass and density functions according to the Bayesian information criteria, and the parameters were fitted simultaneously. Whenever a mixture distribution, the sum of two or more probability functions, was selected, the antimode, the minimum value between two modes of the pdf, was determined (section “Materials and Methods”). The antimode was then used as the threshold to dichotomize the cell population.

**FIGURE 1:**
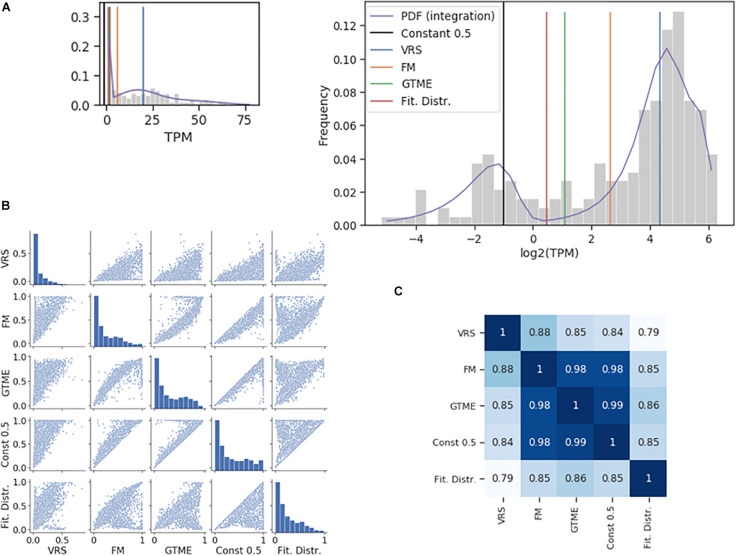
Comparison of dichotomization methods. **(A)** The histogram of the Pcdhac2 transcript numbers in the somatosensory neurons. The dichotomization yielded the following thresholds: 2.11 (GTME), 6.28 (FM), and 20.21 (VRS) TPM. The fitted probability density function (pdf) is a mixture of normal distributions, with an antimode at 1.37. The pdf is integrated piecewise according to the logarithmic bins. The thumbnail plot is a version of the main plot with a linearly scaled *x*-axis. **(B)** Pair-wise scatter plots showing the ON cell frequencies of each gene in the somatosensory neuron dataset with a bimodality coefficient greater than 0.55, after dichotomization with different methods. **(C)** The Spearman rank correlation of the ON cell frequencies shown in **(B)**.

The dichotomization is illustrated using the Pcdhac2 RNA counts from the somatosensory neuron dataset (Figure 1A). The values of the four thresholds differed up to about ten times. The corresponding ON cell frequencies differed less since few cells have RNA counts between the two peaks of the distribution where most of the thresholds are positioned (Figure 1A). Indeed, when comparing ON cell frequencies, all methods were closely correlated (Figures 1B,C); even the lowest correlation had a large value (0.79). For comparison, we also show the dichotomization with a constant threshold at 0.5 TPM. The dichotomization with the antimode correlated most strongly with the GTME-dichotomization (Spearman rank correlation = 0.86), followed by the FM and constant thresholds and last by the VRS (0.79). Therefore, we applied the GTME to all datasets with TPM/FPKM units. It is important to note that GTME thresholds were calculated also for those genes with high bimodality coefficient that yielded unimodal probability density functions, which is often the case, when there are few cells in the OFF or ON expression states (Supplementary Figure 2, Supplementary Table 2, and Supplementary Text 1).

Some datasets had UMI units (Supplementary Table 1). For these distributions, the Bayesian selection and fitting typically returned Poisson or Yule-Simon distributions, and rarely mixture distributions, which precluded the determination of the antimodes. Therefore, we compared the thresholds according to their ability to dichotomize RNA counts of marker genes of specific cell types (Supplementary Figure 3). This led to the selection of the FM-threshold. For most genes, the threshold was positioned between zero and one, simply equating the zero RNA count with the OFF state.

### Effect of Proximity on Stochastic Gene Choice

All RNA distributions were converted into ON cell frequencies with the dichotomization described above. We then examined how proximity affects stochastic gene choice, as genes are often located side by side in gene families with exclusivity. Proximity can influence gene expression in many ways, by promoting the interaction of genes with enhancers via looping, by modifying epigenetic signatures, by relocating chromosomes into active or inactive nuclear compartments, such as transcription factories and heterochromatic compartments (Finn and Misteli, 2019; Monahan et al., 2019).

**FIGURE 2:**
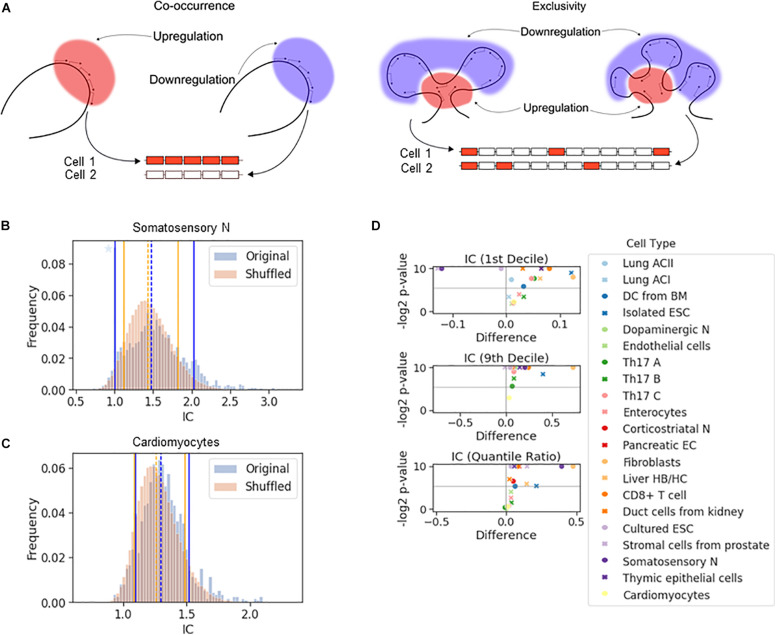
The effect of chromosomal adjacency on stochastic gene choice. **(A)** Schemes showing examples of how the two major forms of stochastic gene choice, concurrence and exclusivity, can arise from alternative chromosomal configurations. **(B,C)** The IC distributions calculated from the original and the shuffled genomes of the somatosensory neurons **(B)** and cardiomyocytes **(C)**. Segmentation size: 14 genes. The blue star denotes a high bar in the histogram hidden by the full line. The location of the 1st (full line), 5th (dashed line) and 9th (full line) deciles is given in the order of original and reshuffled distribution, followed by the *P*-values for the differences: 1.00, 1.48, 2.02; 1.12, 1.44, 1.82; 0.001, 0.016, 0.001. **(B)** 1.10, 1.29, 1.52; 1.09, 1.26, 1.49; 0.217, 0.002, 0.137 **(C). (D)** Volcano plots showing the difference of 1st decile, 9th decile and quantile ratio IC values between the original and the shuffled genomes, along with the corresponding *P*-values (permutation test, segment size: 14 genes). The gray horizontal line at 0.025 corresponds to a two-tailed significance level of 0.05.

If a chromosomal segment shuttles back and forth between sufficiently large active and inactive nuclear compartments, all or none of the genes in that segment will be expressed, which will result in a large cell-to-cell variation in the number of expressed genes in that segment. The all-or-none response is an example of stochastic co-occurrence (a.k.a. concurrence, Figure 2A). In contrast, although each gene is randomly chosen to be expressed, the number of genes expressed in each cell may be the same or similar (Figure 2A, exclusivity). While exclusivity is often equated with the expression of a single gene isoform, this is not necessary as long as the overlap among the chosen genes is small. It is the constant number of expressed isoforms that matters, which is particularly important for protein complexes with fixed stoichiometry. Alternative chromosomal configurations in which a fixed number of genes is located in active nuclear compartments while preventing the remaining genes in the segment from being activated can produce stochastic exclusive gene choice.

The all-or-none response and the fixed, constant number of ON genes in each cell are extreme cases of stochastic co-occurrence and exclusivity, respectively. In this work, we use the terms co-occurrence or exclusivity in a probabilistic (stochastic) sense, and in order to quantify the range of their values, we calculated the interdependence coefficient (IC). IC is the ratio of the cell-to-cell variance in the number of genes chosen to be expressed to the variance of the Poisson-binomial distribution expected from the ON state frequencies of each gene under consideration (see “The Interdependence Coefficient (IC)” in section “Materials and Methods”) (Wada et al., 2018). An IC less than one indicates exclusivity, while an IC greater than one indicates concurrence in stochastic gene choice. When IC is one, the choice of the genes is unbiased, which can reflect independent expression of these genes. Thus, IC enables the detection of exclusive gene choice even when the mean number of expressed genes is greater than one (as in Figure 2A). This illustration shows that the mean number of the ON genes with the exclusive expression can be greater than with concurrent expression (3 versus 2.5), although the variance is significantly lower (0 versus 12).

If a gene affects the probability of the ON and OFF states of the genes in its vicinity, chromosomal segments with exclusive or concurrent expression will be overrepresented. To test this hypothesis, we calculated the IC for segments comprising 14 genes sampled along the chromosomes, which corresponds to the number of genes in the Pcdh-α array. This calculation gives the distribution of the IC values for the original genome. Next, we reshuffled the genes in the genome and calculated the IC for the segments, and by repeating the reshuffling, we obtained a representative distribution of the IC values (Figures 2B,C). To characterize the differences in the distributions, we compared the location of 10th or 90th percentiles (i.e., 1st and 9th decile) to assess the enrichment of the exclusive and concurrent segments, respectively.

In the somatosensory neurons and the prostate stromal cells, the 10th percentile shifted to higher values after the reshuffling, which indicates that the closeness of the genes promotes exclusivity (Figures 2B,D and Supplementary Figure 4A). In some cell types, there is no significant difference in the location of the 10th percentiles (Figure 2C). In the majority of the cell types, exclusivity is suppressed (Figure 2D, top panel). The 90th percentile shifts to substantially lower values when the genome is reshuffled, namely by more than 0.5 in some cells, revealing that all cell types except the prostate stromal cells were enriched in concurrent segments (Figures 2B–D and Supplementary Figures 4A,B). In the original genomes of most cell types, the IC values are more broadly distributed than in the reshuffled genome, as reflected by the quantile ratio of the 9th decile to the 1st decile (Figure 2D, bottom panel), which is mostly due to the overrepresentation of concurrence.

In summary, the permutation tests have shown that the proximity of the genes shifts stochastic gene choice to co-occurrence and suppresses exclusivity in most cell types.

### Stochastic Gene Choice in the Protocadherin Cluster

**FIGURE 3:**
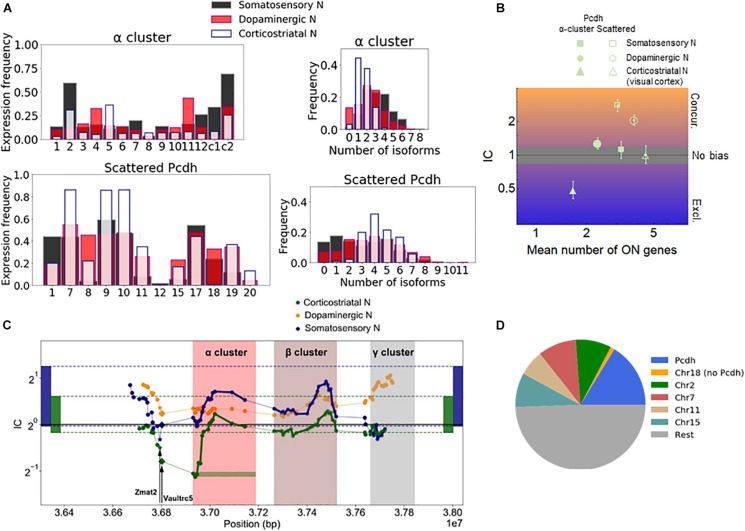
Chromosomal segments with stochastic exclusive gene choice in multiple cell types. **(A)** The expression of the Pcdh α-array and the scattered Pcdhs in different neuronal types. The expression frequency indicates the proportion of cells expressing a particular gene isoform. The distribution of the number of expressed gene isoforms per cell indicates the proportion of cells expressing 0, 1, 2 or more isoforms per cell at the RNA level. **(B)** The IC values calculated from the data shown in **(A)**. The error bars denote the 95% confidence intervals obtained by bootstrapping. **(C)** The IC of segments with 14 genes along the chromosome. The symbols indicate the position of the most upstream gene in each segment. A full segment is denoted by the green horizontal rectangle, at the first gene of the Pcdh-α cluster. The two genes upstream of the Pcdh α cluster (Vaultrc5 and Zmat2) are marked with a star and diamond. The rectangles located at the two extremes of the plot indicate the 2.5 and 97.5 percentiles of the IC distribution calculated for the chromosomal segments in the genome. **(D)** The number of chromosomal segments with exclusive gene choice (as shown in Supplementary Figure 5) in each chromosome for all cell types combined.

The effect of gene proximity can be specifically assessed for the Pcdh family by comparing the Pcdh genes in the α-, β-, and γ-arrays to the Pcdh genes scattered throughout the genome. Most of the scattered isoforms belong to the [δ-protocadherins (Pcdh-1, -7, 8, -9, -10, -11, -17, -18, and -19)] (Redies et al., 2005; Harrison et al., 2020). Especially, the α-array is relevant since the expression there is controlled by chromosomal looping mediated by the CTCF (Jia et al., 2020). The expression of the isoforms varies with the cell type. For example, αC2, α11, and α5 are the most frequently expressed isoforms in the somatosensory, dopaminergic, and corticostriatal neurons, respectively (Figure 3A). The corticostriatal neurons express relatively few α-isoforms with a pronounced exclusivity (Figure 3B and Supplementary Data 1). On the other hand, unbiased choice (or independence, IC not significantly different from one) is observed in somatosensory neurons, and weak concurrence in the corticostriatal neurons.

The α-array can be conveniently compared with the scattered Pcdhs in the somatosensory neurons, as they have a similar number of isoforms, 14 and 12, and the mean number of expressed isoforms is also similar (3.2 and 3.0 genes, respectively). The IC of the scattered isoforms is more than twice as large as the IC of the α-array (Figure 3B). In both the somatosensory and corticostriatal neurons, α-array belongs to the lowest decile of IC distribution. Thus, the α-array in particular gains exclusivity due to the gene adjacency and proximity.

To get a more detailed view of how stochastic gene choice varies along the chromosomal region containing the Pcdh cluster, we moved a 14-gene window along the chromosome to calculate the IC (green horizontal rectangle in Figure 3C). In somatosensory and corticostriatal neurons, the resulting IC profiles are similar along the portion of the chromosome comprising the α- and β-arrays and the region upstream of the array, with the corticostriatal cells having lower IC. The lower IC values in the arrays of the corticostriatal cells can be explained by the lower IC values in the genome when compared to the somatosensory cells, as indicated by the range delimited by the 2.5 and 97.5 percentiles of the genomic IC distribution (Figure 3C).

The above results suggest that IC profiles can be conserved between different cell types. Interestingly, the conserved exclusivity extends upstream of the Pcdh α-array involving the Zmat2 and Vaultrc5 genes (Figure 3C), which suggests that they may be also linked mechanistically and/or functionally to the array. This effect is particularly strong in the somatosensory neurons; in these cells, the segment starting with Zmat2, and comprehending the Vaultrc5 and the 12 variable α isoforms has the lowest IC value altogether in the relevant portion of the chromosome (Figure 3C).

### Chromosomal Segments With Stochastic Exclusive Choice

The above findings suggest that segments with exclusive gene choice can be longer or shorter than previously assumed. To identify chromosomal segments of various lengths that conserve stochastic exclusive expression in multiple cell types, we have segmented the genome into segments comprising 7, 14, or 21 genes. In order to compare different cell types, it is important to take into account that cells in different studies have IC distributions with different mean values (see e.g., Figures 2B,C and Supplementary Figures 4A,B). The difference persists even after the reshuffling, suggesting that it originates from a systemic intrinsic or extrinsic variable. For example, the procedure used for the isolation of cells and RNA and for the RNA detection can introduce positive correlations extrinsically, making the average genomic IC appear larger.

To take into account the above differences, we selected all segments that belong to the lowest 2.5 percentile of the IC distribution in at least two different cell types (or cells cultured in different conditions). We then combined all the segments having 7, 14, or 21 genes that belong to the lowest 2.5 percentile. The two criteria above have been expanded to include a third, stating that the IC must be significantly less than one in at least one of the cell types, i.e., the 95% confidence interval must be below one.

Next, we analyzed the location of these segments. Interestingly, the segments overlapping with the Pcdh array represented the largest fraction (Figure 3D). Segments from the Pcdh array were identified in all analyzed types of neurons (corticostriatal, dopaminergic, and somatosensory), and even in non-neuronal cells, such as endothelial and the lung alveolar cells (Supplementary Figure 5). The Pcdh beta isoforms play a role in tumor suppression in lung cancer (Ting et al., 2019), implying the possibility that exclusive Pcdh expression may diversify cellular identity in non-neuronal cells, as well.

The chromosome 6 harbors a second prominent gene array, the Trbv, which encodes the variable domains of the T-cell receptor. The low IC values of the overlapping segments indicate a strong exclusivity: it is significantly below one in one of Th17 cell variant and numerically less than one in another Th17 cell variant (Supplementary Figure 5). It is important to note that the list of identified arrays with exclusive gene choice is unlikely to be exhaustive because some genes are not detected in a particular cell type. For example, the RNA-seq data cover the expression of Trbv in Th17 cells but not in CD8+ lymphocytes, even though stochastic gene choice and allelic exclusion have been primarily studied in CD8+ lymphocytes. The importance of the exclusivity in T-cell receptor expression in Th17 lymphocytes is underscored by the presence of IL-17 in the cytokine storms, which are thought to contribute to the lethality of the coronavirus disease Covid-19 (Wu and Yang, 2020). Dual reactive lymphocytes that recognize endogenous, neurologically relevant, antigens as well as the coronavirus have also been detected (Boucher et al., 2007).

### Gene Families Shift the Stochastic Gene Choice Toward Co-occurrence

The successful detection of Trbv and Pcdh arrays based on their low IC values indicates that exclusive gene choice can be identified solely based on RNA-seq counts without any information on the alleles and sequence similarity. These gene families have two characteristic features: they are encoded by similar sequences and form an array along the chromosome. The gene family aspect may be more important for the odorant receptors since more than a thousand receptor isoforms are encoded by multiple arrays scattered over a large number of chromosomes. Therefore, after having explored the effect of chromosomal proximity, we turned our attention to gene families.

**FIGURE 4:**
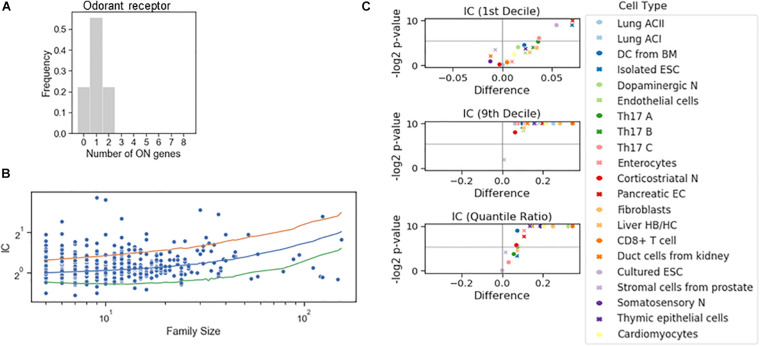
Stochastic interdependence in gene families. **(A)** The number of expressed genes per cell in the family of odorant receptor genes, dichotomized with the familywise threshold (70.7 TPM). Number of cells is *N* = 27. **(B)** IC values of individual families in somatosensory neuron dataset, grouped by the family size. The majority of the families with ICs exceeding either 2.5 or 97.5 IC percentiles of the shuffled genome (orange and green lines, respectively) are concurrent. **(C)** Volcano plots showing the difference of 1st decile, 9th decile and quantile Ratio IC values between the original and the shuffled genomes, along with the corresponding *P*-values (permutation test) calculated for the gene families consisting of 7 genes.

To dichotomize the RNA counts for the gene families, we have not imposed the criterion based on the bimodality coefficient. Instead, we combined the information on the RNA counts of all genes to define the tails of the distribution to calculate a single threshold for all genes in the family. A familywise threshold was used also in a recent study examining how the olfactory receptor expression changes during cell differentiation (Hanchate et al., 2015). We have adapted the GTME to calculate the familywise threshold (fGTME, section “Materials and Methods”). The fGTME threshold resulted in an IC = 0.48 and the mean number of ON genes was 1.0 (Figure 4A), evidencing a marked exclusivity in the choice of olfactory receptors. For comparison, a constant threshold at 0.5 resulted in IC = 3.33 and the mean number of ON genes being around 2 (Supplementary Table 3). Thus, the constant threshold fails to detect the well-established single isoform expression per cell (Supplementary Text 1).

Next, we dichotomized gene expression in each family in various cell types and reshuffled all the genes belonging to a family encompassing at least five genes (Figure 4B). In the somatosensory neurons, there were many gene families with an IC larger than the 97.5 percentile of the IC distribution of the reshuffled genome, but only a few with an IC less than the 2.5 percentile, suggesting that concurrence dominates also in families. Indeed, the systematic examination revealed that the IC at the 10th percentile displayed a significant change in four cell types and the exclusivity was not promoted in any of the cell types. On the other hand, co-occurrence was significantly promoted in all but one cell type (Figure 4C), implying that the shared regulation of the genes in a family shifts gene choice toward co-occurrence.

### The Relation Between Stochastic Gene Choice and Allelic Exclusion

**FIGURE 5:**
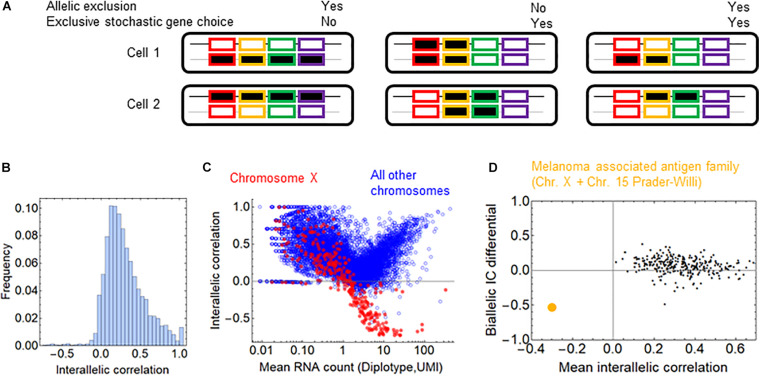
Allelic exclusion and exclusivity in stochastic gene choice. **(A)** Schematic representation of different combinations of exclusivity in allelic and gene choice in an array of four genes. The black and gray lines represent the maternal and paternal chromosomes. The rectangles with no or black filling represent the OFF and ON expression states, respectively **(B)** The interallelic correlation in fibroblasts (Larsson et al., 2019). Negative correlations indicate the allelic exclusion. **(C)** The relation between RNA count and interallelic correlation. The genes on the chromosome X are shown in red. **(D)** The melanoma-associated antigen gene family is highlighted in orange among the gene families. It is the only family with negative mean interallelic correlation.

In addition to the shared regulation of the genes, allelic exclusion may affect stochastic choice in a gene family. The families of the olfactory and T-cell receptors display allelic exclusion, so that only one of the two alleles is expressed, which is also termed monoallellic expression. The molecular mechanisms underlying allelic exclusion can stabilize the gene choice; thus, allelic exclusion may promote stochastic exclusive gene choice. Allelic exclusion takes place after the stochastic choice of the promoter of a T-cell receptor isoform (Ryu et al., 2004). The expression of one allele suppresses the expression of the other allele (Vatakis et al., 2013), a process mediated by various molecular mechanisms. However, allelic exclusion does not necessarily go hand in hand with stochastic gene choice, as the following two examples suggest. Allelic exclusion plays a major compensatory role in the expression of sex chromosomes. In order to compensate for the double dosage of the X chromosomes in females, one of the X chromosomes is inactivated randomly in each cell. Consequently, only one of the gene alleles, the maternal or paternal, is expressed in each cell (Cheng et al., 2019; Zhang et al., 2020); however, this allelic exclusion is not associated with exclusive gene choice because all relevant genes are expressed by one of the chromosomes (Figure 5A). In the protocadherin array, the genes can be expressed monoallelically or biallelically (Kaneko et al., 2006).

To assess whether allelic exclusion can contribute to the choice of gene isoforms on a genomic scale, we analyzed RNA-seq data obtained from heterozygous fibroblasts (Larsson et al., 2019), in which the two alleles of most genes can be distinguished. As a measure for allelic exclusion, we calculated the Spearman correlation coefficient between the two alleles for each gene. The overwhelming majority of the genes displayed positive interallelic correlation. Only a small proportion of genes had negative correlation, most of them are located on the X-chromosome, confirming the predominance of this classical form of allelic exclusion (Figures 5B,C). The allelic exclusion is evident for genes with mean RNA count above 0.5 UMI (Figure 5C). 17 genes from the β- and γ-arrays of the protocadherin cluster are also expressed; all of them have a positive interallelic correlation with a mean value of 0.53 (Supplementary Figure 6A). Next, we calculated two variables for each gene family: the mean value of the interallelic correlations and the biallelic IC differential (see section “Materials and Methods”). The biallelic IC differential is negative if the IC is reduced upon combining the alleles from the two haplotypes, implying that allelic exclusion contributes to exclusivity in stochastic choice in the gene family. Nearly all families have positive mean interallelic correlation, the degree of which does not correlate positively with the biallelic IC differential (Figure 5D). The only gene family with negative mean interallelic correlation is the melanoma associated antigen family. Interestingly, this family experiences the largest shift toward exclusivity in the stochastic gene choice when the two haplotypes are combined: *IC* = 2.47 and 2.67 for the haplotypes and *IC* = 1.52 for the diplotype. Thus, this shift is substantial but not sufficient to attain exclusivity in stochastic gene choice (Supplementary Figure 6B). Most genes of the melanoma associated antigen family are located on the X-chromosome, and the rest of them at the Prader-Willi locus, which is also known to be imprinted (Weon and Potts, 2015; Tacer and Potts, 2017), and explains the marked allelic exclusion in this family. These findings indicate that gene families with allelic exclusion are rare; however, specific gene families can utilize it to enhance exclusivity in stochastic gene choice. Importantly, families with IC less than one have positive mean interallelic correlation (Supplementary Figure 6B), suggesting that stochastic exclusive gene choice does not necessarily imply allelic exclusion.

### Gene Families With Stochastic Exclusive Gene Choice

**FIGURE 6:**
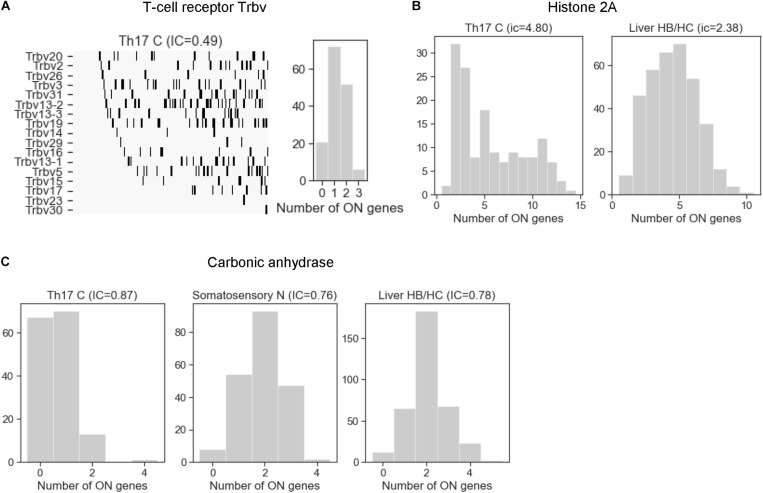
The distribution of the number of expressed gene isoforms (ON genes) per cell in gene families with exclusive and concurrent expression. **(A)** The T-cell receptor beta chain family shows a clear exclusivity in Th17 cells (*IC* = 0.49). The left plot shows the dichotomized expression states. Each column represents a single cell. The gene isoforms are ordered according to expression frequency (highest on the top) and the cells are ordered according to number of expressed isoforms per cell (lowest on the left side). **(B)** The histone 2A family shows co-occurrence in Th17 and liver HB/HC cells, with an IC value of 4.8 and 2.38, respectively. **(C)** The number of expressed carbonic anhydrase genes per cell in Th17 cells, somatosensory neurons, and liver HB/HC (IC = 0.87, 0.76 and 0.78, respectively).

After having analyzed the mechanisms that affect stochastic choice in gene families, we examined exclusivity and co-occurrence in all cell types. The T-cell receptor beta-chain family in the Th17 cells was the most exclusive among all families, with an IC between 0.49 and 0.62 (Figure 6A), comparable to the odorant receptors (Figure 4A). On the other extreme of the scale, the histone 2A family is one of the families with the largest IC values (*IC* = 4.80 in Th17 and 2.38 in liver cells). The histone family nicely illustrates the functional relevance of concurrence: some cells enter the S-phase of the cell-cycle and express the histones to support the ongoing DNA replication, while the cells in the other phases of the cell cycle do not express and/or are degraded (Marzluff and Koreski, 2017), which results in a large coherent cell-to-cell variation in the number of expressed gene isoforms (Figure 6B).

Thus, our analysis with appropriate dichotomization and a simple metric confirmed the exclusive choice in all three prototypic families and gene arrays (T-cell receptor, odorant receptor, Pcdh), so they serve as the positive control for the identification of other gene families. To identify families with stochastic exclusive gene choice, we used the robust approach developed for the chromosomal segments, which combined relative and absolute criteria for exclusivity. The relative criterion ensures that families are selected from the lowest 2.5 percentile of the IC distribution of each cell type. The second criterion states that a family is only considered exclusive if it belongs to the lowest 2.5 percentile in at least two cell types. The last, absolute selection criterion states that the IC must be significantly smaller than one in at least one of the cell types.

**FIGURE 7:**
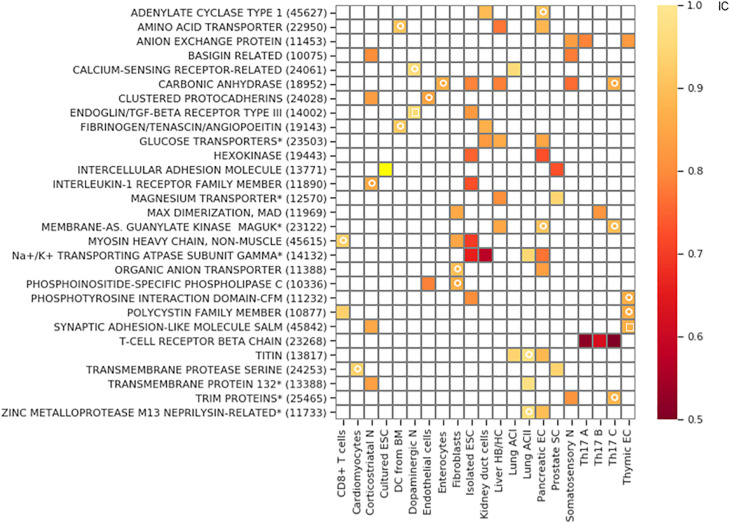
Gene families with exclusive gene choice. Gene families with stochastic exclusive gene choice in two or more cell types; further details of selection as in Supplementary Figure 5 (see also Supplementary Data 1). For the families labeled with star, descriptive names were given instead of the Panther names. The Panther numbers of the families are indicated in parenthesis. The white circle denotes segments with an IC numerically less than 1 without reaching significance. The white empty squares indicates the families that lose exclusivity after truncation of the cell population at the 10th percentile of the total number of detected genes per cell.

The clustered Pcdh family is exclusive in corticostriatal neurons and endothelial cells, and also in the somatosensory neurons. However, in the latter cell type it does not belong to the bottom 2.5 percentile of the IC distribution and consequently, it is not indicated as a hit in Figure 7.

**FIGURE 8:**
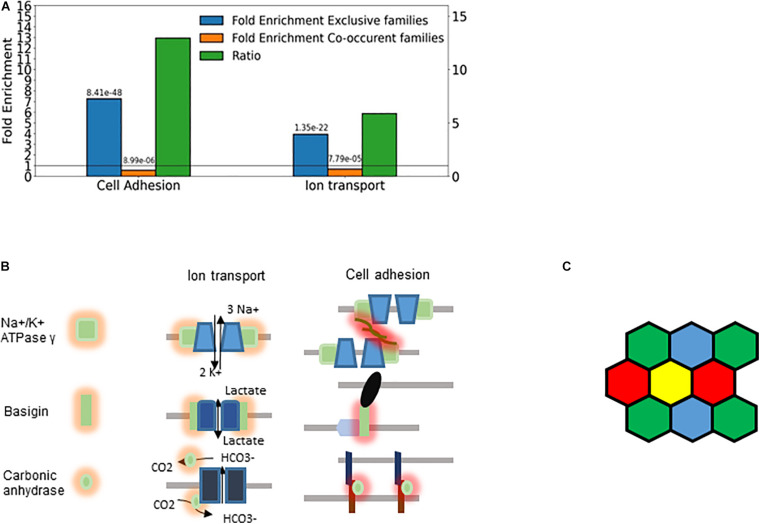
Cellular individuality and cell adhesion. **(A)** Enrichment analysis of the genes belonging to exclusive and concurrent gene families. The *P*-values are indicated on the top of the bars. The exclusive families were selected with the criteria described in Figures 4B, 7. The concurrent families (IC belonging to top 2.5 percentile) were constrained with the following criteria: mean number of expressed genes per cell higher than 0.03, IC significantly higher than 1 and at least 5 non-zero genes per family. We considered all the genes expressed at least in one cell type belonging to the selected families. The enrichment analysis was performed though an enrichment analysis tool (http://geneontology.org/). The figure shows two selected functions: Cell adhesion (GO: 0007155) and Ion transport (GO: 0006811). The ratio of the fold-enrichment in the exclusive to that in concurrent families is shown. **(B)** Schematic representation highlighting the dual role of three gene families (Fxyd, basigin, and carbonic anhydrase genes). On the left side, the cis interaction of the corresponding proteins with channels and pumps is denoted by orange shades. These functions are related to metabolic and ion homeostasis. On the right side, the trans-interaction with ligands on the adjacent cells is labeled with red shades. The glycosylation of the Fxyd protein affects the transdimerization of the Na^+^/K^+^ ATPase. The carbonic anhydrase interacts with the anion exchange protein, which transports HCO_3_^–^. **(C)** Schematic representation of cells showing that the exclusive expression of four gene isoforms (colors) is sufficient to confer cellular individuality in a two dimensional tissue.

The majority of the retrieved families encode membrane proteins (Figure 7) like the three prototypic families. Many of them are associated, directly or indirectly, with two processes: transmembrane ion transport and intercellular adhesion (Figure 8A), These include well-known families involved in cell adhesion such as the basigin related (Bsg, Ccdc141, Cntn5, Cntn6, Dscam, Dscaml1, Emb, Myot, Mypn, Nexn, Nptn, Nrcam, Prtg, Vstm2l) and the synaptic adhesion-like molecule families (Igsf10, Lrfn1, Lrfn2, Lrfn3, Lrfn4, Lrfn5, Lrit1, Lrit2, Lrit3). There are also families primarily involved in ion transport but many of the genes are also involved in cell adhesion, exemplified by the sodium/potassium transporting ATPase subunit gamma and the carbonic anhydrase and anion exchange proteins (Figures 7, 8B).

The Fxyd1-7 gene isoforms encode the gamma subunit of the Na^+^/K^+^ ATPase, which is the regulatory subunit of this ion pump. While these ATPases are primarily involved in ion homeostasis, they can also trans-dimerize and thus mediate cell-to-cell interaction (Tokhtaeva et al., 2016). The stochastic exclusivity of the basigin related genes can be observed in somatosensory and corticostriatal neurons (Figure 7). The members of this family are named after the immunoglobulin−superfamily molecule basigin and are well known mediators of intercellular adhesion (Muramatsu, 2016), comprising genes such as Contactin 6 (Cntn6), Down syndrome cell adhesion molecule (Dscam) and Neuronal cell adhesion molecule (Nrcam). The basigins often interact with monocarboxylic acid transporters, which catalyze the transport of lactate, pyruvate, etc. (Payen et al., 2020); thus, they indirectly affect the ion transport.

The carbonic anhydrase family displays a similar duality of functions related to ion homeostasis and intercellular adhesion, and have a pronounced exclusivity (IC between 0.76 and 0.87; Figures 6C, 7). The primary role of carbonic anhydrases is the catalysis of the reversible conversion of CO_2_ to carbonic acid. However, some isoforms have lost their catalytic activity (Car8, 10, and 11) and they play a role in promoting the diversification in neuronal adhesion and interactions (Sterky et al., 2017).

The analysis of an RNA-seq dataset, which does not meet the inclusion criteria (cells > 100) (Ho et al., 2018), reveals a further gene family involved in cell adhesion, the collagen alpha family, expressed exclusively in corticostriatal and medium spiny neurons (Supplementary Data 2).

### The Efficiency of RNA Detection by Single Cell RNA-seq and Stochastic Exclusivity

**FIGURE 9:**
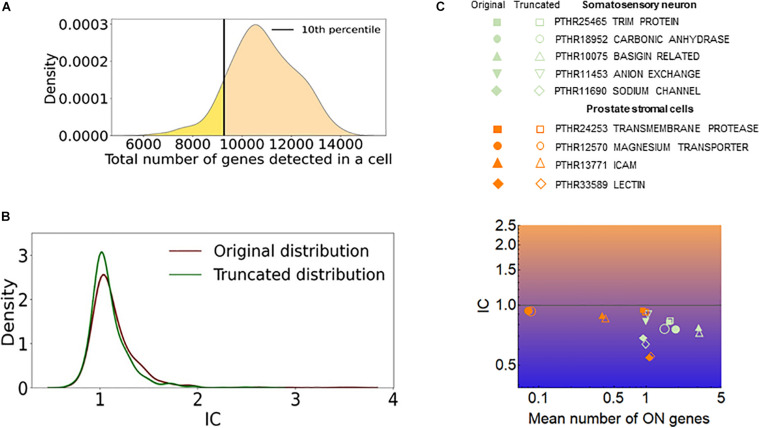
The effect of cells with low number of detected genes on the IC. **(A)** The distribution of the total number of detected genes per cell (dgpc) in the somatosensory neuron dataset. The black line indicates the dgpc below which the cells were removed to obtain the truncated distribution. **(B)** The distribution of IC values of gene families calculated from the original and truncated cell populations shown in **(A)**. **(C)** The IC and the mean number of ON genes calculated with the original (full) and the truncated (empty) datasets. The prostate stromal cell and the somatosensory neuron datasets were used.

The efficiency of RNA detection by RNA-seq is less than 100% and is not uniform in a cell population (Baran-Gale et al., 2018). One may assume that cells with a low number of captured genes mimic exclusivity since only a few genes or gene isoforms are detected in these cells. To assess how such a cellular heterogeneity affects the quantification of stochastic gene choice, we removed 10 percent of the cells with the lowest number of detected genes and calculated the IC from the truncated population (Figure 9A). If the removed cells were accountable for exclusivity, the truncation would have increased the IC. However, the mean IC did not increase; in fact, it decreased slightly in the truncated population of the somatosensory neuron dataset and also in all other datasets (Figure 9B and Supplementary Table 4). Figure 9C shows the exclusive gene families with the lowest IC in the prostate stromal cells and the somatosensory neuron datasets, which have the lowest and highest numbers of detected genes per cell, respectively. The amiloride-sensitive sodium channel family (PTHR11690) has the lowest IC in the somatosensory neurons, whereas the PTHR33589 in the prostate stromal cells, which includes Jacalin-like lectin domain-containing proteins. The exclusive families detected in two cell types are also displayed. After truncation, the mean number of ON genes increases in most of these families, as expected, since cells with a low number of genes are removed. Importantly, the IC remained less than one in all of the families, and in several cases the IC even decreased after the truncation. Similarly, the IC remained less than one in all but two exclusive families shown in Figure 7.

Six datasets with TPM units having the largest gene coverage (above 8,000, see Supplementary Table 4) yield 34 hits while the remaining 11 TPM datasets yield only 27 hits. Thus, lower gene coverage in these datasets does not seem to lead to spurious hits, but rather reduces the success rate of the detection of exclusive families. Accordingly, the development of newer single cell RNA-seq technologies with higher capture efficiency may enable the detection of more families with exclusive gene choice.

## Discussion

### Determinants of Exclusivity in Gene Families and Chromosomal Segments

Our results show that stochastic exclusivity is rare in both gene families and segments and concurrence is overrepresented. Multiple mechanisms are likely to underlie this phenomenon. Evolving from a single gene, paralogs have common regulatory sequences. Consequently, a shift from concurrence toward exclusivity is expected only after a sufficient evolutionary divergence in the family. Chromosomal proximity can also promote concurrence when a transcription factor affects multiple genes in a chromosomal segment (Wada et al., 2019). For example, two copies of the same gene at the same chromosomal position experience more correlated fluctuations if they are positioned on linked chromosomes than on physically separated, but homologous, chromosomes (Becskei et al., 2005). Furthermore, the positive correlation in stochastic gene expression has gradient-like features along the mammalian chromosomes (Sun and Zhang, 2019). Thus, the predominance of concurrence in the genome can be viewed as a direct consequence of evolutionary-genetic and biophysical-chemical processes.

Despite the dominance of concurrence in chromosomal segments, chromosomal proximity may promote exclusivity in the appropriate context. A single gene in the Pcdh α-genes can be chosen to be expressed upon the formation of a CTCF-mediated chromosomal loop between the chosen gene and a downstream enhancer (Wu Q. et al., 2020; Wu Y. et al., 2020). This looping mediated gene proximity may promote exclusivity and may explain the much higher exclusivity of the Pcdh α-array in comparison to the scattered Pcdhs. Recent findings indicate that the arrangement of CTCF binding sites as tandems play an important role since they insulate gene expression and thus effect stochastic promoter choice (Jia et al., 2020).

Exclusivity has no general molecular marker for all three classical exclusive gene families. Variations even exist among the Pcdh arrays. CTCF controls the expression of the Pcdh β-isoforms, as well (Hirayama et al., 2012; Sams et al., 2016) but the β-array has a larger IC than the α-array (Supplementary Data 1). Furthermore, cell-specific mechanisms are likely to explain why the expression in the Pcdh-α array is exclusive in some neuronal types but unbiased in others (Figure 3B). It is also possible that the interactions of the neurons during development determine whether or not stochastic gene choice is exclusive, which means that gene expression and cell adhesion are under mutual control.

The calculation of and analysis with IC has multiple advantages. It can help to define the range of chromosomal segments subject to exclusive gene choice, especially when the genes do not belong to a family. For example, the exclusivity in the α-Pcdh array extends beyond the array and affects two upstream genes, Zmat2 and Vaultrc5. Zmat2 has been shown to regulate the splicing of genes involved in cell adhesion (Tanis et al., 2018). Thus, Zmat2 may directly affect the Pcdh-mediated cell adhesion. Furthermore, Vaultrc5 is a vault RNA, which controls autophagy, and several Pcdh proteins are known to associate with autophagy related proteins (Buscher et al., 2020).

Similarly, the IC formalism does not require predefined sets of genes for the assessment of exclusivity. For example, the αC1 and αC2 isoforms are usually excluded from the analysis when the number of expressed gene isoforms is quantified in the α-array due to their constitutive expression in Purkinje cells (Esumi et al., 2005). However, their expression is not constitutive in other cell types: the αC1 and αC2 isoforms are expressed at a lower frequency than some of the variable isoforms (α1-12) in corticostriatal neurons (Figure 3A). Since the IC formalism does not assume a single gene to be expressed in order to be exclusive, it permits the detection of exclusivity in all these cell types with different mean number of expressed genes.

IC has another important aspect, the absolute value. The T-cell receptor family with IC values as low as 0.5 has an unmatched degree of exclusivity in comparison to the other detected exclusive families. This may reflect the fact that multiple different molecular mechanisms cooperate to stabilize exclusive stochastic gene expression: the promoter choice through chromosomal looping is followed by DNA recombination and allelic exclusion (Massah et al., 2015). DNA recombination is unlikely to contribute to the exclusivity in the families involved in cell adhesion. The exact mechanism underlying exclusivity, looping or covalent epigenetic modifications or other processes, remains to be determined (Magklara and Lomvardas, 2013; Almenar-Queralt et al., 2019).

### Functional Relevance of Stochastic Exclusive Gene Choice

We have used relatively stringent criteria to identify families with exclusive choice since they had to be detected in at least two different cell types. Despite the overrepresentation of concurrence in most genomes, the exclusive gene choice is not restricted to the T-cell receptor, odorant receptor and Pcdh families. Ten other families were identified with pronounced exclusivity, with IC less than 0.8: the anion-exchange and basigin related proteins, the carbonic anhydrases, intercellular adhesion molecule, interleukin-1 receptor family, phospholipase C, the sodium/potassium transporting ATPase gamma subunit, the hexokinases and the non-muscle myosin heavy-chain. Most of them directly affect cell adhesion (Figure 7), but even hexokinases can affect motor or cytoskeletal proteins, and thus regulate cellular adhesion (Hsu et al., 2010; Ghosh et al., 2016). Ion transport is the second most overrepresented function in the detected families. Ions have been long known to modulate cell adhesion (Arcangeli and Becchetti, 2006). In addition to calcium, magnesium and pH are of major physiological relevance in cell adhesion (Takeichi and Okada, 1972).

Ion transport and cell adhesion can be regulated by the same protein (Figure 8B). For example, the ratio of the Fxyd5 isoform to the α1–β1 heterodimer determines whether the Na^+^/K^+^ ATPase acts as a positive or negative regulator of intercellular adhesion (Tokhtaeva et al., 2016). This is highly reminiscent of the Pcdh proteins, in which the ratio of the expressed isoforms determined intercellular adhesion (Yagi, 2012; Thu et al., 2014). Interestingly, basigin can also bind the β2−subunit of Na^+^/K^+^ ATPase (Heller et al., 2003).

The carbonic anhydrase isoforms Car10 and Car11 are secreted glycoproteins that are predominantly expressed in the brain. Car10 was shown to be a conserved pan-neurexin ligand (Sterky et al., 2017). Neurexins, like protocadherins, mediate interneuronal interactions, but the isoform diversity is generated primarily through alternative splicing (Mauger and Scheiffele, 2017) and not by stochastic gene choice. Overexpression of Car10 in neurons creates a shift in neurexin isoforms in mouse and human neurons, which may explain how the stochastic choice of Car isoforms generates diversity. Even catalytic Cars affect intercellular adhesion. For example, Car9, a cancer associated transmembrane isoform of carbonic anhydrase, reduces E-cadherin mediated adhesion (Svastova et al., 2003). The Cars can interact with the anion exchange proteins, Slc4a, which transport bicarbonate (Morgan et al., 2007), which is thought to accelerate CO_2_ transport. Thus, two families with exclusive expression can interact physically. It remains to be determined how gene families involved in glucose transport and metabolism profit from exclusive expression. Recent advances in the description of the spatial variations in metabolism across a cell-population (Ben-Moshe and Itzkovitz, 2019; Polyzos et al., 2019) do suggest that not only cell adhesion but also ion homeostasis may profit from stochastic exclusive gene choice. The transmembrane serine proteases (Tmprss) may also affect cell adhesion by regulated proteolysis, which can help cancer cells to spread (Qiu et al., 2007; Tanabe and List, 2017).

Cells interact through homophilic or heterophilic interactions (Ahrens et al., 2002; Thu et al., 2014; Brasch et al., 2018). The affinity of the interaction can depend on the particular combination of the respective protein isoforms (Yagi, 2012). Thus, diversity through gene choice can have functional consequences. For example, choosing two isoforms from a repertoire of five genes permits 10 combinations, and thus 10 cellular identities. It is important to note that cells in a plane can become fully distinguishable with the exclusive expression of four different gene isoforms, according to the four color theorem (Figure 8C; Wu et al., 2015). Somewhat higher numbers are needed for cells arranged in 3-dimensional interaction networks. Thus, the detected families with 5 or more members are in principle capable of supporting sufficient diversity to enable each cell to distinguish itself from its neighbors.

The combinatorial diversity due to the random choice of multiple gene isoforms is translated into a diversity of cell-to-cell interactions, while the exclusivity guarantees the precise stoichiometry within the membrane protein complexes. This principle is a conserved property of many gene families involved in cell adhesion and ion transport beyond the protocadherins, suggesting that stochastic exclusive gene choice is an ideal mechanism to link diversity with precision in cell adhesion.

## Materials and Methods

### Data Sources

To define the chromosomal segments, the Genome Reference Consortium Mouse Build 38 patch release 6 (GRCm38.p6) was used^1^. The genes marked as predicted were excluded, and only the genes sourced from Best-placed RefSeq (BestRefSeq) and Curated Genomic were considered.

PANTHER15.0 was used to map genes to their corresponding gene families^2^ (Mi et al., 2019).

The single cell RNA-seq datasets are described in Supplementary Table 1.

### Interconversion of RNA-Seq Quantification Units

TPM (Transcripts Per Million) units were analyzed without conversion. The RPKM (Reads Per Kilobase Million) and FPKM (Fragments Per Kilobase Million) can differ between samples, causing biases for the statistical interpretation of the data (Wagner et al., 2012). Therefore, they were converted into TPM units (Kim et al., 2018):

$$TPM_{g}=\frac{FPKM_{g}}{\sum_{j}FPKM_{g}}10^{6}$$

*FPKM*_*g*_ represents the FPKM values of a given gene. The gene counts are summed over the population of *j* cells.

Datasets with Unique Molecular Identifier (UMI) counts were used without further normalization.

### Dichotomization of Expression Into ON and OFF States for the Genes in the Chromosomal Segments

To exclude the genes with unimodal expression, the bimodality coefficient was calculated for each gene:

$$b=\frac{g^{2}}{k+\frac{3{\left(n-1\right)}^{2}}{\left(n-2\right)\left(n-3\right)}}$$

where *k* is the sample excess kurtosis, g is the sample skewness, n is number of samples (i.e. cells) (Knapp, 2007). Only the genes with *b* > 0.55 were kept since a value of 5/9 or less corresponds to a unimodal distribution. This filtering was applied to data in TPM units for the analyses of chromosomal segments.

Three methods were compared to dichotomize the expression of individual genes: VRS, FM and GTME. The minimum threshold was set to be 0.5 TPM, which is widely used as threshold for a gene considered to be expressed. Thus, when a procedure resulted in a threshold with a value less than 0.5 TPM, it was replaced by 0.5 TPM. Upon determining the threshold, the genes are dichotomized. If the expression value is greater than or equal to a threshold, the gene is marked as expressed in this cell (i.e., with 1), otherwise it is marked as not expressed (i.e., with 0).

#### Variance Reduction Score (VRS)

VRS is a measure of bimodality, in that it reflects how much the variance of the original distribution is reduced in comparison to the sum of the variances of the two distributions obtained by the splitting of the original distribution with a threshold (Hellwig et al., 2010).

$$VRS=\frac{\sum_{x\in X_{below}}{\left(x-{\bar{x}}_{below}\right)}^{2}+\sum_{x\in X_{above}}{\left(x-{\bar{x}}_{above}\right)}^{2}}{\sum_{x\in X}{\left(x-\bar{x}\right)}^{2}}$$

where *X* is a total set of expression values of a gene, *X*_*below*_ and *X*_*above*_ are sets of expression values lower than and greater than or equal to a threshold, respectively. $\bar{x}$, ${\bar{x}}_{below}$ and ${\bar{x}}_{above}$ are the mean expression values for the three sets, respectively.

In order to find the threshold with the minimal VRS, a range of threshold values were tested for each gene. This range is a list of geometrically progressing series with the step of 1.2 starting at 0.025 quantile of non-zero expression values up to the 0.975 quantile to get a more granular view of VRS at lower thresholds. The threshold that yields the minimum VRS is chosen as a dichotomization threshold.

#### Fraction of Maximal Values (FM)

The FM is a biochemically motivated threshold and assumes that the expression of a gene does not vary too much around its activity specific to the ON state. For this purpose, the 1/10th of the TPM value at the 97.5 percentile was chosen. If the number of cells with non-zero expression values (*N*) is less than 120, then the 1/10th value of the average (arithmetic mean) of the three largest values was calculated.

$$FM=\left\{ \begin{array}{c} \frac{x_{0.975}}{10},N\geq 120\\ \frac{\sum_{i=N-2}^{N}x_{i}}{10},N<120 \end{array}\right.$$

where *x*_*p*_ is the *p*th quantile of non-zero expression values, *x*_*i*_ is the *i*th element of the sorted non-zero expression value list, *N* is the number of non-zero expression values,

#### Geometric Trimmed Mid-Extreme (GTME)

The GTME is motivated by the predictions of transition rates in bistable systems: the threshold between the two states is defined as the geometric mean of the low and high states (Hsu et al., 2016). Bistable systems can underlie bimodal distribution but there is no simple relation between them because of the transiency (Pajaro et al., 2019). In order to define the threshold without knowing the exact values of ON and OFF states, the geometric mean of the non-zero TPM values at the bottom and top 2.5 percentiles (40-quantiles) of the distribution were taken. If the number of non-zero TPM values is less than 120, the average (arithmetic mean) of the three least and largest values were used to calculate the geometric mean.

$$GTME=\left\{ \begin{array}{c} \sqrt{x_{0.025}\cdot x_{0.975}},N\geq 120\\ \sqrt{\sum_{i=1}^{3}x_{i}\cdot \sum_{i=N-2}^{N}x_{i}},N<120 \end{array}\right.$$

Analogous thresholds allow for the precise calculation of the transition rates in a bistable cell population (Hsu et al., 2016).

### Familywise Thresholds

Assuming that the expression values of genes within a family are similar, a common threshold can be defined for all genes within a family. The familywise FM (fFM) and GTME (fGTME) were calculated as follows. The RNA counts larger than 0.5 were considered instead of the x > 0 condition. When the respective cell number *N* was larger than 120, the *x*_*g*, 0_._025_ and *x*_*g*, 0_._975_ were calculated for each gene. The fFM was calculated from the maximum of the set of *x*_*g*, 0_._975_, *g* ∈ *GF*, representing each gene in a gene family (*GF*). Thus, a single gene in the family determines the threshold for all the genes in the family. Similarly, the two genes corresponding to the minimum of the *x*_*g*, 0_._025_ and the maximum of the *x*_*g*, 0_._975_ *g* ∈ *GF*, set determine the fGTME. Analogous calculation were performed for *N* < 120, with mean averages of the three largest and smallest expression values, instead of the values at the percentiles.

### Fitting of Distributions

Probability density (or mass) functions, *φ(x*), were fitted with the FindDistribution of Wolfram Mathematica, which combines the Bayesian information criterion with priors over distributions to select both the best distribution and the best parameters for it. Commonly fitted distributions were the Binomial, Cauchy, Exponential, Gamma, Geometric, Normal, Laplace, Logistic, Lognormal, Poisson, Negative Binomial, Yule-Simmons distribution and their mixtures. Whenever a mixture distribution was obtained by the FindDistribution, the antimodes were calculated. The antimodes were determined analytically based on the first and second derivatives of *φ*(*x*). The smallest antimode in the range *x* > 0.5 was used as thresholds for dichotomization for each gene. As opposed to other methods, the *φ*(*x*) based thresholds were not used for calculation of IC across the genome, since they were obtained for a smaller number of genes in comparison to the other methods. This is because the fitting of *φ(x*) is less robust, especially when there are few cells in the OFF or ON expression states or when the measurement error is larger.

### The Interdependence Coefficient (IC)

The IC is the ratio of the observed variance in the number of expressed genes in a cell population to the variance of the Poisson binomial distribution expected from the expression frequencies (Wada et al., 2018). The variance of the generalized binomial (Poisson-binomial) distribution is a function of the probability of each isoforms *i* to be expressed (*p*_*i*_):

$$IC=\frac{\sigma_{OBS}^{2}}{\sigma_{PB}^{2}},\text{where}\sigma_{PB}^{2}=\sum_{i=1}^{N_{\alpha }}\left(1-p_{i}\right)p_{i}$$

*p*_*i*_ is equal to the ON cell frequency. IC = 1 indicates an unbiased (independent) stochastic gene choice according to the Poisson-binomial distribution, akin to a relation Fano-factor = 1, which indicates a Poisson distribution for a single gene (Ozbudak et al., 2002).

The 95% confidence interval (CI) of the IC was calculated by bootstrapping. After resampling the cell population, the observed variance and the expected Poisson-binomial variance were calculated for each resampling, and IC was calculated. When the 95% CI was below one, exclusivity was considered significant.

### Permutation Tests

Permutation tests were used to assess the effect of chromosomal adjacency and family membership on stochastic interdependence. The expression values of the genes are shuffled among all genes but for those that were not measured in a particular dataset or were not bimodal. The shuffling was performed 1000 times. Similarly, the assignment of genes (i.e., their respective expression values) to gene families is shuffled. Only the genes that are present in both the families and the RNA-seq datasets are reassigned in a way that the sizes and number of families are preserved. The distribution of IC values were obtained for each re-shuffling.

The 10th and 90th percentiles and their ratio were calculated as representative quantiles for the exclusivity and concurrence. Therefore, the *P*-values for the changes in the quantiles were calculated based on the permutation tests (Ernst, 2004). The *P*-value was calculated as follows

$$Pvalue=\frac{1+\sum_{i=1}^{N}I\left(|\hat{x}-\bar{x}|\geq |x_{i}-\bar{x}|\right)}{1+N}$$

where $\hat{x}$ is the original statistic, $\bar{x}$ is the mean of the shuffled statistic, *x*_*i*_ is the statistic of the ith permutation, and *N* is the number of permutations. The pseudocount is added to avoid *P*-values of 0. Since 1,000 permutations were performed, the smallest *P*-value is 0.001.

A two-tailed *P*-value of 0.05 was selected for a statistic to be considered significantly higher or lower than the statistic of the shuffled distributions. Exclusivity is promoted when the 10th percentile of the original distribution is significantly smaller. Similarly, co-occurrence is promoted when the 90th percentile is significantly greater. These tests were applied for each chromosomal segment size separately. Families were grouped according to their size, and the same tests were performed as for the chromosomal adjacency. Only family sizes that have 30 or more gene families were taken for the permutation tests.

### Identification of Genes Subject to Concurrent or Exclusive Gene Choice in Multiple Cell Types

To assess which sets of genes conserve their mode of interdependence across multiple cell types, the pairwise overlap of gene segments or gene families that are within the bottom or top 2.5 percentiles of their respective IC distributions was determined. In other words, a segment or a family is considered a hit, if it appears in two datasets in the respective tails of IC distributions. The chromosomal segments were overlapped separately for each segment size, whereas all families were considered together (Supplementary Data 1). Further conditions to filter the selected genes are described in the relevant context.

### Examination of the Relations Between Allelic Exclusion and Stochastic Gene Choice

The mean interallelic correlation was calculated by averaging the Fisher transform of the Spearman correlation coefficient calculated for the two alleles, followed by a back transformation (Alexander, 1990):

$$\bar{\rho_{S}}=Tanh\left[\frac{1}{N}\sum_{i=1}^{n}Arctanh\left[\rho_{Si}\right]\right]$$

To calculate the biallelic IC differential between the diplotypes and haplotypes, the following formula was used:

$$\mathrm{Biallelic}\mathrm{IC}\mathrm{differential}=\mathrm{Log}\left[\frac{IC_{Diplotype}}{\sqrt{IC_{Haplotype\_1}IC_{Haplotype\_2}}}\right]$$

## Data Availability Statement

The algorithms used in this work are available in the GitHub repository: https://github.com/d-lowl/stochastic-gene-choice.

## Author Contributions

AB designed the project and wrote the manuscript. MI wrote the programs. MI, SF, and AB analyzed the data. All authors contributed to the article and approved the submitted version.

## Conflict of Interest

The authors declare that the research was conducted in the absence of any commercial or financial relationships that could be construed as a potential conflict of interest.

## Footnotes

1 https://www.ncbi.nlm.nih.gov/assembly/GCF_000001635.26

2 ftp://ftp.pantherdb.org/sequence_classifications/current_release/PANTHER_Sequence_Classification_files/PTHR15.0_mouse
