# Supplementary material for: Gene Families With Stochastic Exclusive Gene Choice Underlie Cell Adhesion in Mammalian Cells
Source: Front Cell Dev Biol. 2021 Apr 29;9:642212. doi: 10.3389/fcell.2021.642212 (PMC8117012; doi:10.3389/fcell.2021.642212)
Supplement: Supplementary file 1 [file Data_Sheet_1.pdf]

Supplementary Material

Supplementary Text 1

Supplementary Figures 1 to 6

Supplementary Tables 1 to 4

Supplementary Data 1 to 2

Supplementary Text 1. Comparison of the dichotomization methods.

The overview describes the design of the dichotomization and related data processing described in Supplementary Table 2. The detailed procedures and formulas are described in the Materials and Methods. At the end, we compare the dichotomization of genes in families using the fGTME and the constant 0.5-threshold.

We use the following nomenclature for the terms related to stochastic gene choice. Gene expression in single cells as measured by RNA-seq is a discrete or continuous variable, which is dichotomized to obtain ON and OFF expression states. Thus, stochastic gene choice refers to genes with ON and OFF expression states. The IC is calculated form the dichotomized data (0, 1). Stochastic gene choice can be exclusive, independent or concurrent according to the value of the IC.

Screening for bimodality with the bimodality index (BI)

For the analysis of chromosomal segments, a restriction (BI > 0.55) was imposed only to the datasets with TPM units but not with the UMI units. This difference originates from the fact that fitting of the UMI distributions resulted only rarely in mixture distributions, which have the potential to have a minimum between the two peaks.

For the analysis of the gene families, the bimodality index was not applied at all since the information of all families is combined and thus, even unimodal distributions can be assigned to the ON or OFF states correctly with high certainty.

Minimal threshold (0.5)

For FPKM/ TPM units, 0.5 is commonly considered as a cutoff, and the following specifications are used for the expression levels: cutoff (0.5), low (0.5 to 10), medium (11 to 1000), high (1000 and more) [https://www.ebi.ac.uk/gxa/FAQ.html].

For each type of analysis and dataset, a specific method was used to determine the threshold (Supplementary Table 2). Whenever the calculated threshold was below 0.5 it was replaced by 0.5 Thus, the minimum threshold for all methods was set to 0.5. This minimum threshold filters out spurious peaks.

For example, the dichotomization of the somatosensory neuron dataset resulted in 6673 0.5 thresholds out of the 20854 measured genes. Thus, 32% of the thresholds is at 0.5 TPM.

In the datasets with UMI units, all data less than one are zeros. Thus, a 0.5 value is symbolically chosen, as an arbitrary value below one.

Selection of thresholds and their validation

For the chromosomal segments, as described in the main text, the antimode of the probability mass/density function was used to validate the thresholds for the TPM datasets whereas the accuracy of the dichotomization of the marker genes was used to validate thresholds for the UMI datasets.

To design the dichotomization for the gene families, we adapted the thresholds definitions for the segments to the families with the respective units (Supplementary Table 2).

For the chromosome segments calculated with TPM data sets, the constant threshold value of 0.5 correlates almost as well with the antimode dichotomization as the GTME (Figure 1). Analyzing the UMI records with the constant threshold results in less accuracy than FM and GTME, but higher accuracy than VRS. (Supplementary Figure 3). Thus, GTME and FM are among the two best methods for analyzing TPM and UMI datasets.

Minimal number of genes in a segment to calculate IC

To ensure sufficient diversity in the expressed genes, each segment or family had to contain a minimal number of genes.

1. *Chromosomal segments*

Each chromosomal segment had to contain a minimum number of genes that were measured in a particular dataset and that were bimodal (TPM).

This minimum number was 6, 12 and 18 for the segments encompassing 7, 14 and 21 genes, respectively.

2. *Gene families*

Each family had to contain at least five genes for the calculation of the IC. The gene families were then ranked according to the IC values. Gene families belonging to the bottom 2.5 percentile of the IC distribution were then processed by the overlap analysis. In the overlap analysis, additional criteria were introduced to identify gene families with exclusive gene choice in multiple cell types. The number of genes with larger than zero ON cell frequencies had to be 5 or 6 depending on whether the IC was significantly below one or only numerically.

Validation of the fGTME on classic exclusive gene families and comparison to the constant threshold value of 0.5

The dichotomization by the fGTME plays a central role in our study since it is used to identify gene families with exclusive gene expression given the fact that the majority of datasets have or were converted to TPM units.

Therefore, the fGTME was additionally validated by comparing to the classic exclusive families expressing a single gene (Tcrbv, olfactory receptor). Here, we compare the dichotomization with a constant threshold value of 0.5 for all gene families.

The fGTME correctly identifies strong exclusivity in both families as well as the mean number of expressed genes, is in line with previous expectation (Supplementary Table 3). On the other

hand, the constant 0.5 threshold yields inconsistent results. For example, olfactory neurons results in a large IC indicating concurrence (IC = 3.33) instead of the marked exclusivity (0.48). Furthermore, the mean number of expressed genes increases from one to two. For the T-cell receptor family Trbv, both the constant 0.5 and the family-wise thresholds yield exclusive gene expression. However, the mean number of expressed genes is considerably larger with the constant threshold (around 2 versus 1.3). The latter number is more realistic since 3 to 10% of T-cells have bialleic Tcrbv recombination (2), which would result in a mean number of expressed isoforms of around 1.1 (Tcrbv isoforms per cell at the RNA level). Furthermore, if the 0.5 threshold is applied, the Pcdh α-array will be not exclusive in any of the cell types (Supplementary Data 1). These examples show that familywise thresholds are superior to the constant thresholds in the detection of gene families with exclusive gene choice.

REFERENCES

1. Baran-Gale J, Chandra T, Kirschner K. Experimental design for single-cell RNA sequencing. Brief Funct Genomics. 2018;17(4):233-9.
2. Brady BL, Steinel NC, Bassing CH. Antigen receptor allelic exclusion: an update and reappraisal. J Immunol. 2010;185(7):3801-8.

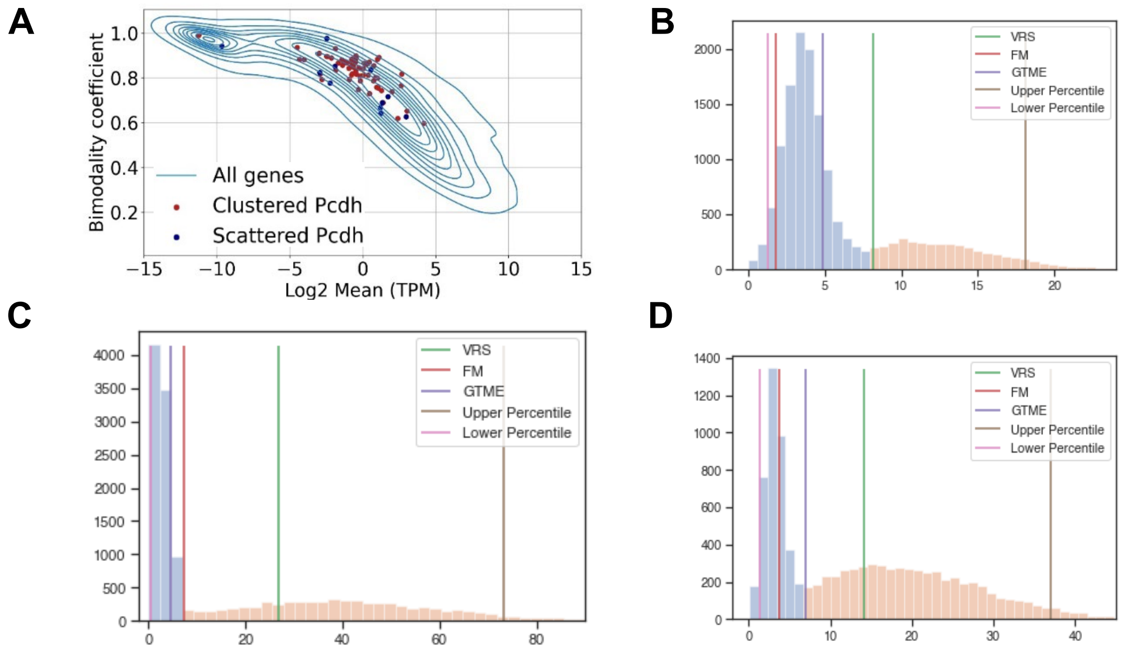

Supplementary Figure 1. Bimodality and thresholds to dichotomize the distributions. **(A)** The bimodality index (*b*) calculated for the genes the somatosensory neuron dataset. All genes are shown as isobaric density plots, whereas the Pcdh genes are also indicated in form of scatter. There is an anticorrelation between the mean TPM and *b*; the Spearman rank correlation coefficient (all genes) is -0.88. **(B-D)** Simulated bimodal distributions representing mixtures of two normal distributions *N*(mean, standard deviation). The blue and orange parts denote the distributions left and right to the antimode, respectively. Varying the parameters of the distributions, the best threshold is obtained by different dichotomization methods. (B) *N*(3.5, 1.2) and *N*(11, 5) with a 2:1 proportion. (C) *N*(2, 2) and *N*(38, 22); 10:7. (D) *N*(3, 1) and *N*(17, 11); 3:7.

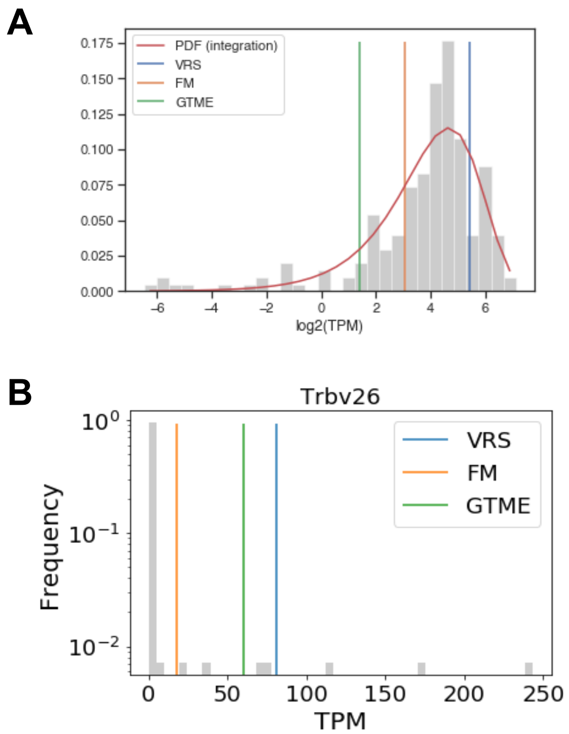

Supplementary Figure 2. RNA counts fitted with distributions without antimodes. (**A**) The Piezo2 gene is expressed in all cells of the cell population. An exponential function is fitted with lambda = 0.039. The thresholds yield the following ON cell frequencies: 0.17 (VRS), 0.76 (FM) and 0.90 (GTME) TPM. The plot on the right side is the version of the main plat with a linearly scaled x-axis. (**B**) The expression of Trbv26 in Th17 cells. BI = 0.88. The majority of cells do not express this gene and a Poisson distribution with a mean value close to zero is fitted (not shown).

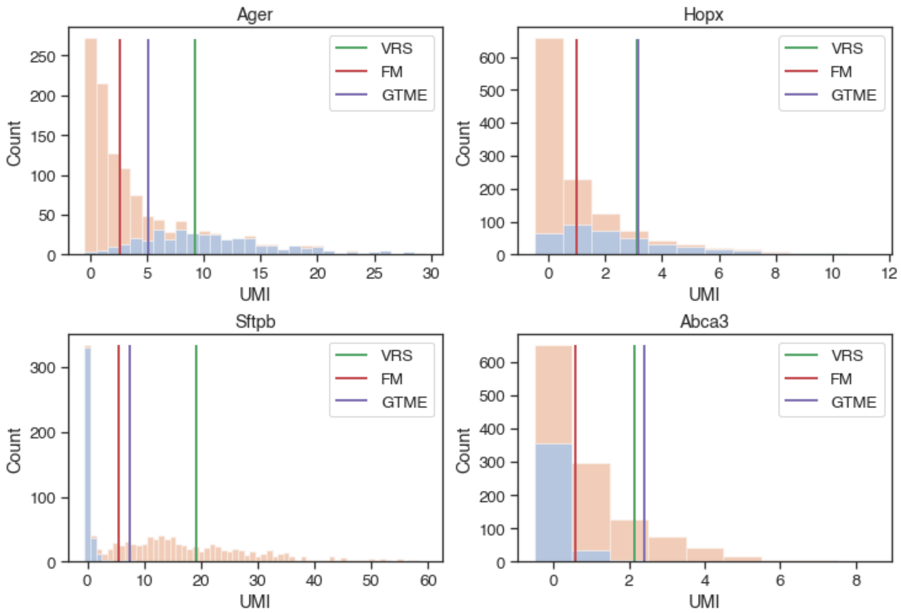

	VRS	FM	GTME	0.5
**Ager**	0.84	0.80	0.90	0.54
**Hopx**	0.75	0.75	0.75	0.75
**Sftpb**	0.61	0.93	0.90	0.94
**Abca3**	0.44	0.72	0.44	0.72
**Mean Accuracy**	**0.66**	**0.80**	**0.75**	**0.74**

Supplementary Figure 3. The histograms show the expression of four marker genes in Type I (blue) and Type II (orange) alveolar cells. 0.5 denotes a constant threshold at 0.5 UMI, which separates zero from non-zero counts. The Table below contains the accuracies. FM is associated with the lowest misclassification of the two cell types, as evidenced from the highest mean accuracy yielded by this method.

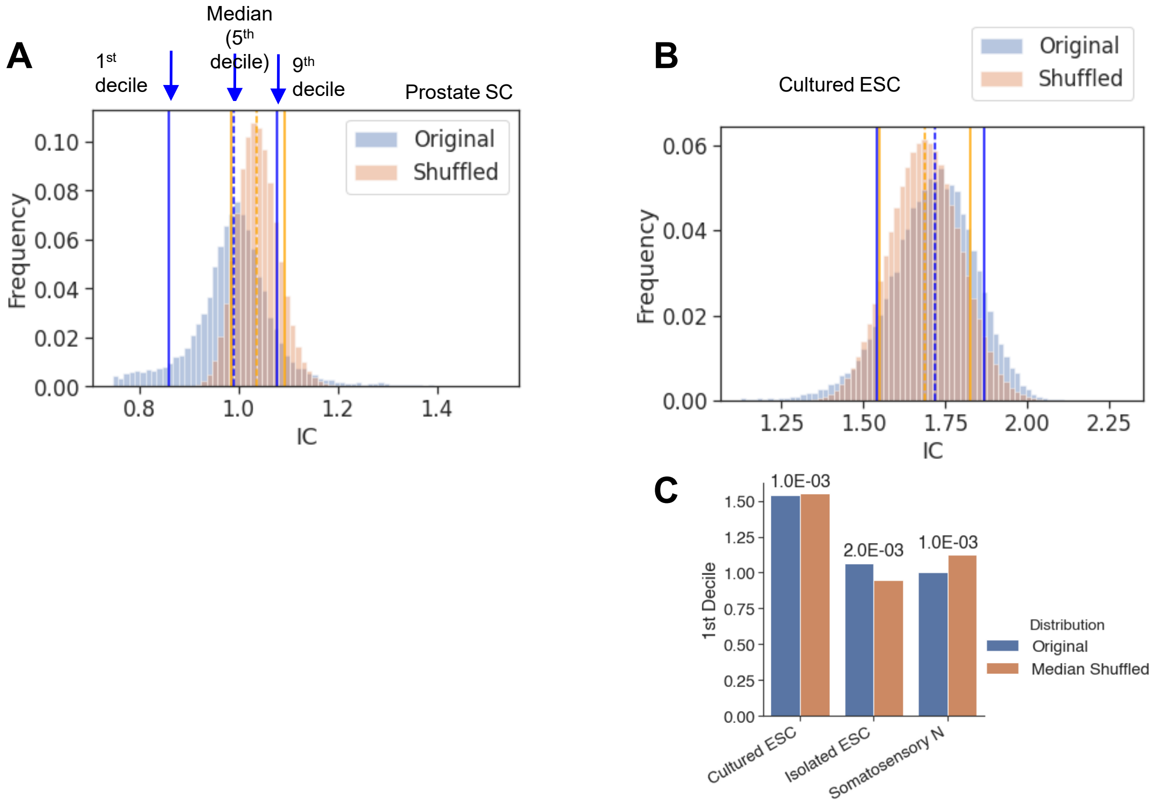

Supplementary Figure 4. Effect of the chromosomal proximity on the stochastic interdependence analyzed by the permutation test. (**A, B**) The location of the 1st (full line), 5th (dashed line) and 9th (full line) deciles is given in the order of original and reshuffled distribution, followed by the P-values for the differences: 0.85, 0.99 and 1.08; 0.99, 1.04 and 1.09; 0.001, 0.001 and 0.001 (A) 1.54, 1.72 and 1.87; 1.55, 1.69 and 1.83; 0.001, 0.001 and 0.001 (B). (**C**) The 1st decile IC values are shown for the original and the shuffled genomes, along with the P-values (permutation test) for the differences in the three cell types.

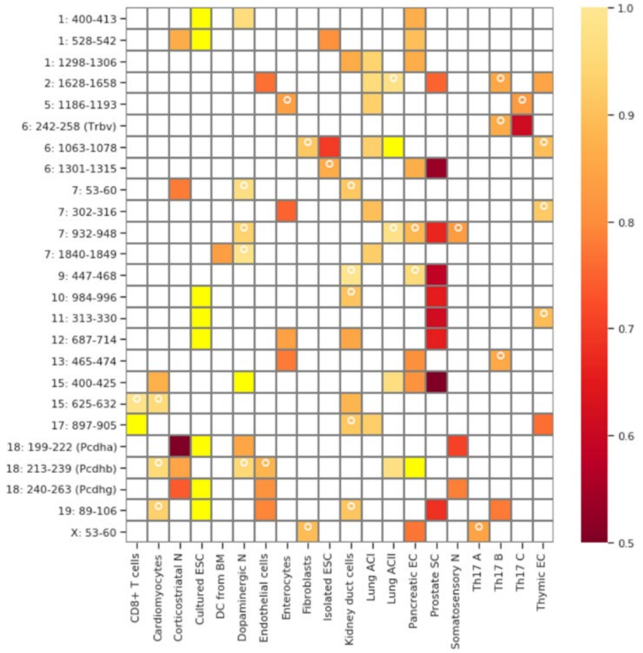

Supplementary Figure 5. Chromosomal segments with IC values in the bottom 2.5 percentile of the IC distributions in more than two cell types. The segments contain 7, 14 or 21 genes. Segments having an IC value significantly less than one at least in one cell type are shown provided they express at least 0.03 genes per cell on average and at least 5 gene isoforms in the population of a particular cell type. The white circle denotes segments with an IC numerically less than 1 without reaching significance. In this case, 6 gene isoforms had to be expressed in a cell type. The yellow squares denote IC values greater than one which still belong to the bottom 2.5 percentile. The Tcrbv family is not shown in the Th17A cells because only 11 genes in the 14-gene segment were measured and dichotomized.

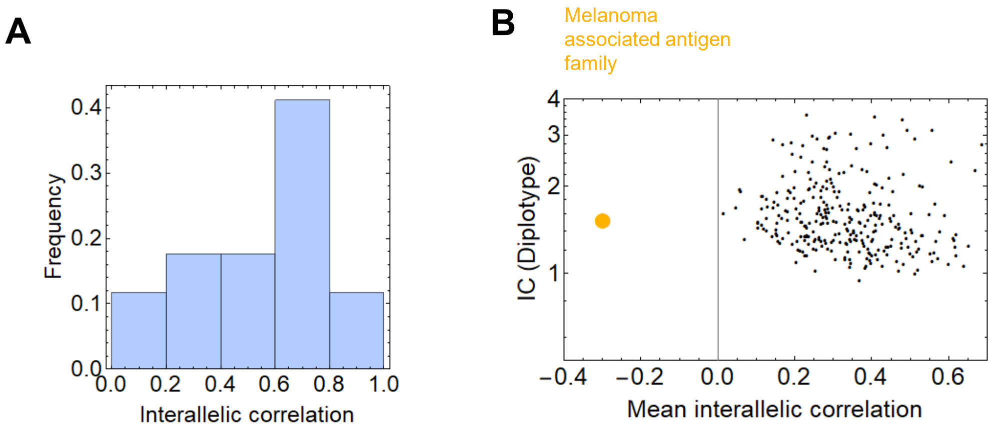

Supplementary Figure 6. The relation between allelic exclusion and the interdependence coefficient. **(A)** Interallelic correlation in the Pcdh genes expressed in fibroblasts. The beta and gamma isoforms are expressed. **(B**) The relation between interallelic correlation and IC for gene families. The Spearman rank correlation between these two variables is -0.23 (P=0.000086).

Supplementary Table 1. Description of RNA-seq data

Cell type	Number of cells	Sequencing platform (mRNA count format, seq. library)	Mouse line [Age]	Author (Accession number)
Somatosensory neurons (N) from DRG	204	Illumina HiSeq 2500 (FPKM, SMARTer Ultra Low)	C57BL/6 [8-10 weeks]	*Li et al.* (GSE63576)
Corticostriatal neurons (N)	190	Illumina HiSeq 2500 (TPM, SMARTer Ultra Low)	C57BL/6J [P56±3]	*Tasic et al.* (GSE71585)
Dopaminergic neurons (N)	473	Illumina HiSeq 2500 (FPKM,Smart-Seq2)	Th:EGFP BAC transgenic mice (Tg(Th-EGFP)DJ76Gsat/Mmnc) [E15.5 and P7][1]	*Hook et al.* (GSE108020)
Bone Marrow (BM) dendritic cells (DC) progenitors	251	Illumina HiSeq 2000 (RPKM, C1 Fluidigm)	C57BL/6 [7-10 weeks]	*Schlitzer et al.* (GSE60781)
Cardiomyocytes	482	Illumina HiSeq 2500 (RPKM, Smart-seq2)	C57BL/6 [8 weeks]	*Nomura et al.* (GSE95140)
CD8+ T cells	287 [2]	Illumina HiSeq 2500 (TPM, SMARTer)	C57BL/6J	*Kakaradov et al.* (GSE89405)
Cultured ES cells (ESC)	935	Illumina HiSeq 2500 (UMI, CEL-SEQ)	IB10 (derived from 129/Ola strain)	*Klein et al.* (GSE65525)
Embryo-isolated ES cells (ESC)	1724	Illumina HiSeq 2000 (RPKM, Smart-Seq2)	Different mouse lines. [E5.25, E5.5, E6.25 and E6.5] [3]	*Cheng et al.* (GSE109071)
Endothelial cells[4]	111	Illumina HiSeq 3000 (FPKM, SMARTer Ultra Low)	C57BL/6J [6-10 weeks]	*Veerman et al.* (GSE106514)
Enterocytes from intestinal epithelium	155[5]	Illumina NextSeq 500 (TPM, SMART-Seq2)	C57BL/6 [7-10 weeks]	*Haber et al.* (GSE92332)

Fibroblast	354[6]	Illumina HiSeq 2000 (RPKM, Smart-seq2)	CAST/EiJxC57BL/6J or C57BL/6JxCAST/EiJ	*Reinius et al. (*GSE75659*)*
Kidney duct cells (DC)	235[7]	Illumina HiSeq 3000 (TPM, SMART-seq)	C57BL/6 [2 months]	*Chen et al. (*GSE99701*)*
Liver Hepatoblasts / hepatocytes (HB/HC)	353	Illumina HiSeq 2000 and 2500 (TPM, Smart-Seq2)	F1 progenies of C57BL/6 and C3H (B6C3F1) mice [E10.5 to E17.5][8]	*Yang et al. (GSE90047)*
Lung alveolar cells (types I and II) (ACI, ACII)	1222 (395 AT1, 827 AT2)	Illumina HiSeq 2500 (UMI, Drop-seq)	C57BL/6J [P1]	*Guo et al. (GSE122329)*
Mature thymic epithelial cells (EC)	174	Illumina HiSeq 2500 (FPKM, SMART-seq)	C57BL/6 [4 weeks]	*Sansom et al. (GSE60297)*
Pancreatic endocrine cells (EC)	438	Illumina HiSeq 4000 and 2500 (TPM, Smart-Seq2)	C57BL/6 [E10.5 to E12.5]	*Yu et al. (GSE115931)*
Prostate stromal cells (SC)	1417	Illumina HiSeq 2500 (FPKM, iCELL8)	C57Bl/6 [8-10 weeks]	*Craighton et al. (GSE119988)*
Th17 cells (Th17 A., Th17 B., Th17 C)[9]	139 (Th17 A), 130 (Th17 B), 151 (Th17 C)	Illumina Genome Analyzer II (FPKM, SMART-Seq)	C57BL/6	*Gaublomme et al. (GSE74833)*

[1] Th-eGFP+ dopaminergic neurons collected at two timepoints from three brain regions (midbrain, forebrain, olfactory bulb).

[2] One cell from this dataset was excluded from the analysis since it showed no expression for the whole gene set.

[3]There are three types of mice crossing: 1) C57BL/6J (C57) female mice mated to C57 male mice; 2) CAST/EiJ (CAST) female mice mated to C57 male mice; 3) C57 female mice mated to CAST male mice.

[4] The analysed data comprise blood endothelial cells and high endothelial cells.

[5] Different cell types have been analysed in the original paper, included enterocytes, enterocytes progenitors, enteroendocrine cells, goblet cells, paneth cells, TA cells and Tuft cells; for this work, only enterocytes have been considered.

[6]Split-cell lysates and pooled cells have not been considered.

[7]Different cell types have been analysed, included Proximal tubule cell, intercalated cells and principal cells.

[8]The whole set of cells of this study is composed of 447 hepatoblasts/hepatocytes and 82 cholangiocyte. Some of these cells have been treated with molecules to accelerate the differentiation process. We selected the untreated cells identified as hepatoblasts/hepatocytes (353 cells).

[9] Three different growth protocols; IL1B_IL6_IL23-48h-IL-17A/GFP+, TGFB1_IL6-48h and TGFB1_IL6-48h-IL-17A/GFP+ (139, 130 and 151 single cells).

Supplementary Table 2. Steps of data processing for the dichotomization to analyze chromosomal segments and gene families.

**Type of Analysis**	**Chromosomal segments**		**Gene families**	
**Data type**	**FPKM or TPM**	**UMI**	**FPKM or TPM**	**UMI**
Data conversion	FPKM→TPM	None	FPKM→TPM	None
Screening for bimodality with bimodality index	genes with B.I. > 0.55	No	No	No
Threshold type	GTME	FM	Family-wise GTME (fGTME)	Family-wise FM (fFM)
Minimal threshold	0.5	0.5	0.5	0.5
Selection of thresholds	Correlation to antimode of the fitted probability density function	Accuracy of the dichotomization of marker genes	Adaptation of the threshold for segments (GTME) to the families.	Adaptation of the threshold for the segments (FM) to families.
Minimal number of measured and dichotomized genes in a segment / family to calculate IC.	6 genes in a 7-gene segment,<br>12 genes in a 14-gene segment,<br>18 genes in a 21-gene segment.		Number of genes with positive ON cell frequencies:<br>5 genes when the IC is significantly below 1<br>6 genes when the IC is numerically below 1<br>(These conditions were applied to detect families with exclusive expression with the overlap analysis)	
Minimal mean number of ON genes (gene isoforms) per cell			0.03 for the overlap analysis to identify gene families with exclusive gene choice	

Supplementary Table 3. The mean number of ON genes and the IC of representative families calculated with constant and geometric dichotomization.

	**Family-wise threshold (fGTME)**			**Constant threshold (0.5)**		
**Gene family (cell type)**	Threshold (TPM)	Mean number of ON genes	IC	Threshold (TPM)	Mean number of ON genes	IC
**Trbv Th17A**	86.6	1.15	0.52	0.5	1.80	0.56
**Trbv Th17B**	192.2	1.34	0.62	0.5	2.02	0.67
**Trbv Th17C**	83.1	1.28	0.49	0.5	1.80	0.54
**Olfactory rec. Olfactory neurons**	70.7	1.00	0.48	0.5	1.97	3.33
**Histones 2A Th17A**	35.4	5.42	5.38	0.5	16.93	4.06
**Histones 2A Th17B**	34.1	8.28	5.91	0.5	17.54	3.38
**Histones 2A Th17C**	37.8	5.73	4.80	0.5	16.95	4.47

Supplementary Table 4. The total number of detected genes per cell (dgpc). A gene is considered detected if it has a TPM (or UMI) > 0. The IC is calculated based on the original distribution and the distribution truncated at the 10th percentile of the original distribution.

Cell type	Number of cells	Mean dgpc	dgpc 10th Percentile	dgpc 90th Percentile	Mean IC (original)	Mean IC (trun-cated)	IC Ratio (orig. / trunc)
**Cardiomyocites**	482	6215	4110	7843	1.16	1.10	1.06
**CD8+ T cells**	287	7076	5563	8654	1.43	1.42	1.01
**Corticostriatal N**	190	8706	7580	9727	1.08	1.06	1.02
**Cultured ESC**	933	11997	8504	15059	1.66	1.48	1.11
**DC from BM**	251	3997	2885	5296	1.09	1.08	1.01
**Dopaminergic N**	473	5644	2949	7669	1.35	1.22	1.09
**Endothelial cells**	111	5598	4521	6733	1.12	1.11	1.00
**Enterocytes**	155	6103	4959	7098	1.11	1.11	1.01
**Fibroblasts**	354	8374	7440	9382	1.20	1.16	1.03
**Isolated ESC**	1724	9250	8064	10501	1.17	1.15	1.02
**Kidney duct cells**	235	6075	4264	7921	1.12	1.11	1.01
**Liver HB/HC**	353	8882	7874	9702	1.18	1.15	1.02
**Lung ACI**	395	1074	611	1668	1.13	1.13	1.01
**Lung ACII**	827	1349	687	2058	1.20	1.18	1.01
**Pancreatic EC**	438	8016	6641	9495	1.23	1.22	1.01
**Prostate SC**	1417	892	263	1433	1.02	1.01	1.01
**Somatosensory N**	204	10854	9269	12640	1.14	1.10	1.04
**Th17 A**	139	7800	6754	8754	1.09	1.08	1.01
**Th17 B**	130	8331	7319	9145	1.15	1.15	1.00
**Th17 C**	151	7729	6669	8715	1.09	1.08	1.01
**Thymic EC**	174	5228	3792	6509	1.17	1.15	1.01
